# A call for methodological rigour: suspending systematic reviews and meta-analyses of observational studies on ultra-processed foods

**DOI:** 10.1038/s41430-026-01764-9

**Published:** 2026-05-19

**Authors:** Jimmy Chun Yu Louie

**Affiliations:** Department of Allied Health, School of Health Sciences, Swinburne University of Technology, Hawthorn, VIC Australia

**Keywords:** Nutrition, Epidemiology

## Introduction

The proliferation of systematic reviews and meta-analyses (SLRMAs) examining ultraprocessed foods (UPFs) and health outcomes, e.g. [1–3], has generated substantial scientific and public interest, yet these syntheses have amplified rather than resolved uncertainty. The fundamental challenge transcends individual study quality: a profound methodological incompatibility exists between conventional dietary assessment tools and the conceptual framework required to evaluate UPF consumption [4]. This mismatch creates systematic exposure misclassification that undermines the validity of individual studies and, consequently, any attempt to synthesize their findings [4–6].

When researchers retrospectively apply Nova to dietary data collected using tools not designed for this purpose, they must make subjective coding decisions based on incomplete information. These decisions often vary substantially between research groups, and even across individuals within the same research group, generating inconsistency across entire food categories rather than merely for isolated items [5, 7]. Most countries lack standardized Nova classification databases [4], forcing researchers to make individual judgments that may not align with Nova protocols or with decisions made by other research teams investigating similar questions.

The problem persists even when meta-analyses examine specific foods within the UPF category rather than aggregating all UPF together, the underlying measurement problem remains unresolved. Dietary assessment tools still lack the granular detail necessary to determine whether items such as chocolate or bread belong in particular processing categories. When studies use instruments not designed for processing-based classification and are compelled to make assumptions when retrospectively coding ambiguous items, these misclassifications become embedded in study results. Pooling these potentially flawed estimates in meta-analyses produces summary estimates that may reflect methodological artifacts rather than genuine biological relationships [8]. The statistical precision achieved through meta-analytic techniques creates an illusion of scientific rigor that obscures underlying measurement problems [9].

This *Perspective* addresses the methodological challenges of synthesizing observational evidence when dietary exposure is assessed retrospectively with tools not intended for UPF classification. While acknowledging that randomized controlled trials on UPF have been conducted [10–13] and alternative systems have been proposed [14], these fall beyond the scope. RCTs typically control the foods provided [15, 16], thereby avoiding the retrospective classification issues that complicate observational studies, while some newer systems may be less affected by these limitations than Nova, as they draw on different types of information [14], some of which are already captured in conventional dietary assessment tools [17].

## Methodological foundations and their limitations

Observational UPF studies rely predominantly on dietary assessment instruments developed decades before the UPF classification system was conceptualized. Food frequency questionnaires (FFQs), the most common dietary assessment method in epidemiological studies, were designed to assess frequency of consumption of predefined food items to estimate nutrient intake and identify dietary patterns based on conventional food groups [18]. These tools ask respondents to report how often they consumed items such as “bread” or “chicken” over a specified time period [17], but they were typically not designed to probe the specific details about food sourcing, ingredient composition, preparation methods, or presence of cosmetic additives that are essential for accurate Nova classification [4, 5].

This represents not an oversight or omission but a fundamental limitation of the FFQ format, which prioritizes feasibility and respondent burden over the granular detail that processing-based classification demands. Similarly, 24-h dietary recalls and food records, while potentially capturing more detail than FFQs, still rarely systematically collect the information necessary to distinguish between foods in different Nova categories [4, 5]. Because the concept of UPF and the Nova system only emerged around 2010 [19], they were never incorporated into the design of tools used in large epidemiological studies launched in earlier decades [5].

Consider a “chicken sandwich” reported in a 1990s FFQ. Its classification depends on details that cannot be determined from the available data [4]: preparation method, ingredient sourcing, presence of “cosmetic” additives, and whether it was homemade or store-bought [5, 20]. Applying Nova classifications to such historical data requires retrospective coding that introduces considerable uncertainty, yet this uncertainty is rarely quantified or adequately acknowledged in study conclusions

The study by Sneed et al. [21]. examining reliability (whether researchers consistently apply Nova to the same foods) has shown high agreement between trained coders, but this consistency does not address validity when the underlying dietary data lack the information necessary for accurate classification. Researchers may consistently classify “bread” as Nova-4 while having no information about whether it was homemade, artisan-produced with minimal ingredients, or industrially manufactured with multiple additives and preservatives. Consistency of misclassification does not resolve the fundamental problem of insufficient information in the source data.

Substantial variation exists in how different research groups classify the same foods. In one study [22], homemade bread and cured meats that were explicitly described by Nova’s creator as processed (Nova-3) [20], were instead coded as ultra-processed (Nova-4). Similar discrepancies have been documented across multiple food categories and between different countries [4, 5]. When meta-analyses pool results from studies with such fundamental inconsistencies in exposure classification, the resulting summary estimates become profoundly difficult to interpret.

## The compounding effect of evidence synthesis

SLRMAs are predicated on the assumption that individual studies provide reliable estimates of exposure-outcome relationships that can be meaningfully combined [23, 24]. However, when the underlying exposure measurements are fundamentally flawed, the resulting pooled estimates represent artifacts of methodology rather than genuine insights into biological relationships [8].

Prospective cohort studies face particular challenges in maintaining exposure measurement validity over extended follow-up periods [25]. Dietary patterns evolve, food products are reformulated, and new products enter the market [25]. The assumption that baseline dietary assessments accurately reflect habitual intake throughout the follow-up period becomes increasingly tenuous as the duration of observation extends [17]. This temporal misalignment is particularly problematic for UPF research, given the dynamic nature of food processing technologies and the continuous evolution of the food supply [26, 27].

Over recent decades, food manufacturers have reformulated existing products, introduced new product lines, and modified processing techniques in response to consumer demands, regulatory changes, and technological advances [26, 27]. Some changes have improved nutritional quality, such as reductions in *trans* fats or sodium [28], while others have increased the degree of processing or introduced new types of additives [26, 29]. For studies that assessed diet in the 1990s but analysed health outcomes through 2020, the foods consumed at baseline may no longer exist in their original formulation, or they may now be categorized differently under Nova if their ingredient lists or processing methods have changed. This temporal disconnect between exposure measurement and outcome assessment introduces a layer of uncertainty that meta-analyses cannot resolve through statistical aggregation.

## Implications for scientific communication and policy

Meta-analytic findings on UPFs have often been presented in public health messaging and policy recommendations with levels of certainty that are unsupported by the underlying methodology. Recognising these pre-existing concerns, journals and editors-in-chief should consider appending a methodological caveat to already-published UPF SLRMAs, clearly communicating to readers that the validity of the underlying exposure measurements is substantially limited.

Media coverage of meta-analyses frequently report strong associations between UPFs and health outcomes using definitive language (e.g. [30],), without acknowledging the methodological limitations regarding exposure measurement [5]. The inventor and supporters of the Nova system have argued that evidence from SLRMAs justifies immediate policy action, e.g. [20, 31–36], despite the significant measurement concerns that affect the validity of the underlying studies.

This disconnect between methodological reality and public messaging creates an inflated sense of evidentiary strength. Policies based on UPF classification, when built on evidence from studies with fundamental measurement problems, risk focusing public health resources on an imprecisely defined exposure category rather than on specific dietary patterns or nutrients with clearer evidence of harm. Policymakers who have already incorporated UPF SLRMAs into regulatory or dietary guidance frameworks are urged to exercise particular caution when interpreting these findings, given that the evidentiary base may not yet support the degree of certainty with which it has been communicated.

While processing may indeed be relevant to health outcomes, premature policy action based on methodologically compromised evidence may prove less effective than interventions targeting well-established dietary risk factors such as excessive sodium [37], saturated fat [38], or sugar-sweetened beverages intake [39]. The imperative for evidence-based public health demands that policy recommendations reflect the actual strength and limitations of available evidence rather than aspirational certainty.

## Recommendations for the research community

### Immediate actions and conditional framework

Here I am proposing that an immediate moratorium be placed on the publication of SLRMA of observational studies examining UPFs and health outcomes. This moratorium should remain in effect until substantial improvements in dietary assessment methodologies are achieved and validated. Should the research community determine that UPF SLRMA must proceed despite these recommendations, journal editors, peer reviewers, and authors conducting SLRMAs must implement fundamental changes to the review methodology.

Any future SLRMA examining UPFs must incorporate mandatory dietary assessment evaluation criteria that extend beyond traditional risk of bias instruments to include specific domains addressing dietary assessment validity [40]. Each included study should be evaluated for the appropriateness of the dietary assessment method for UPF classification. This evaluation should examine whether the instrument collected sufficient detail on food sourcing and preparation methods, brand names, and ingredient compositions, and the granularity of food types covered, particularly for FFQs. Studies should also be assessed for temporal alignment between tool development and implementation of processing-based classification systems, and for transparency in handling ambiguous food items.

Studies using tools not designed for processing-based classification should receive lower quality ratings, with this limitation reflected in overall certainty assessments. While emerging frameworks for assessing risk of bias in systematic reviews include domains relevant to exposure measurement [40], most existing instruments do not adequately address the specific challenges of UPF classification applied retrospectively to data from conventional dietary assessment tools. Applying such criteria would result in systematic downgrading of evidence quality for studies lacking appropriate exposure measurement, which would in turn reduce the certainty of pooled estimates and more accurately communicate the substantial uncertainty inherent in current evidence syntheses on this topic.

These enhanced quality assessment protocols must be accompanied by comprehensive discussion requirements for authors and editorial oversight from journals. Any SLRMA must address dietary assessment limitations across included studies, potential impact of exposure misclassification on pooled estimates, heterogeneity in food classification approaches between studies and countries, and implications for interpretation of findings. While many published studies acknowledge limitations related to dietary assessment in their discussion sections, generic acknowledgment does not adequately address the fundamental problem when measurement tools are genuinely inappropriate for the research question.

Assertions that results remain valid despite acknowledged limitations require empirical support, such as validation studies demonstrating that the classification achieves adequate accuracy despite imperfect measurement, or sensitivity analyses showing that results are robust to plausible levels of misclassification. In the absence of such evidence, claims of validity represent unjustified optimism rather than scientific rigor. SLRMAs must therefore include explicit acknowledgment of the speculative nature of retrospective food classification when applied to data collected using tools not designed for this purpose, and must clearly communicate that the scope of measurement problems in this literature exceeds what can be adequately addressed through limitations statements alone.

### Long-term methodological development and institutional reform

The research community should focus on developing dietary assessment tools specifically designed to measure UPF intake, with validation against objective indicators of food processing and tested for reliability in diverse populations. Several recent critiques have similarly called for methodological improvements in UPF research [4–6, 41], with O’Connor, Herrick et al. [4] highlighting fundamental challenges in measuring UPF exposure using conventional dietary assessment methods.

Building on these important contributions, new tools must include detailed questions about food preparation methods, ingredient lists, brand names, and purchase locations, all of which are essential for accurate processing-based classification. While some existing methods such as detailed food records and multiple 24-h dietary recalls can theoretically capture this level of detail [17], most large epidemiological studies examining UPF have not employed these intensive assessment approaches [4, 5]. Even when these more detailed methods are used, the information collected often remains insufficient for confident processing-based classification, particularly when data were gathered before such classification systems were conceptualized. The critical distinction is that new tools must be designed from the outset with processing-based classification as the primary objective, ensuring that all necessary information is systematically captured rather than relying on retrospective inference from data collected for other purposes.

Validation studies must go beyond comparing one self-report measure against another and instead use objective data, such as food packaging, purchase receipts, or biomarkers. Biomarkers represent a promising avenue for more objective assessment of UPF intake and could help validate self-report measures or serve as independent indicators of exposure. While exploratory work has begun to identify potential biomarkers associated with UPF consumption [42], substantial validation is needed to establish whether specific biomarkers can reliably distinguish processing-based categories from other dietary factors such as overall diet quality or specific nutrient patterns. Although newer tools designed to capture processing characteristics have begun to appear in the literature [43–45], some rely on flawed validation methods, such as comparing a Nova-based FFQ with a traditional one [46], both of which share the same underlying limitations.

The development of standardized classification databases across countries and regions is equally important. These databases should include clear protocols for assigning processing-based categories, systems for handling new or reformulated products, and processes for resolving classification disagreements. To improve accuracy and reduce misclassification, technology-based tools such as mobile apps, barcode scanners, and image-assisted dietary assessments should be rigorously tested against both traditional and objective methods. Training programs for researchers must also be strengthened, with clear guidance on managing ambiguous cases and implementing quality control procedures to ensure consistent and reliable processing-based classification across studies.

Institutional changes are needed to prevent the continued production and promotion of methodologically inadequate research on UPFs. Studies that cannot validly measure UPF intake due to flawed dietary assessment tools, inappropriate use of UPF classification systems, or insufficient methodological detail should not be funded or published. These studies contribute noise into the evidence base, distort conclusions, and undermine confidence in nutrition science. Journals must strengthen editorial standards, requiring transparent, technically sound methods for classifying and assessing UPF intake. Peer review panels should include at least one expert in dietary assessment. Funders should require clear justification for how UPF exposure will be measured and should reject proposals that rely on incompatible or poorly suited datasets. Professional societies must establish minimum methodological standards to guide both primary research and evidence reviews in this area. Supporting methodologically inadequate studies delays progress and contributes to confusion in public health messaging.

## Conclusion

The current approach to synthesizing observational research on UPFs reveals a profound mismatch between the complexity of the questions being asked and the limitations of the available methods. Publishing SLRMAs that rely on studies with inadequate exposure measurement does little to clarify the science. Instead, it adds to the confusion and damages the credibility of nutritional epidemiology.

The proposed moratorium on these analyses should be understood as a measure of quality control, not suppression. It reflects a commitment to grounding conclusions in sound methodology rather than presenting a false sense of certainty through the aggregation of methodologically inadequate data. Nutritional research should aim to produce reliable evidence that can genuinely inform public health guidance and individual choices. Until the core methodological issues in UPF research are resolved, the field should avoid generating SLRMAs that overstate what the data can actually support. This represents not a retreat from scientific inquiry but rather an advancement toward more rigorous, transparent, and ultimately more useful nutritional science.
